# Variable control of the electronic states of a silver nanocluster *via* protonation/deprotonation of polyoxometalate ligands†

**DOI:** 10.1039/d2sc01156e

**Published:** 2022-04-12

**Authors:** Kentaro Yonesato, Seiji Yamazoe, Soichi Kikkawa, Daisuke Yokogawa, Kazuya Yamaguchi, Kosuke Suzuki

**Affiliations:** Department of Applied Chemistry, School of Engineering, The University of Tokyo 7-3-1 Hongo Bunkyo-ku Tokyo 113-8656 Japan ksuzuki@appchem.t.u-tokyo.ac.jp kyama@appchem.t.u-tokyo.ac.jp; Department of Chemistry, Graduate School of Science, Tokyo Metropolitan University 1-1 Minami Osawa Hachioji Tokyo 192-0397 Japan; Graduate School of Arts and Science, The University of Tokyo 3-8-1 Komaba Meguro-ku Tokyo 153-8902 Japan; Precursory Research for Embryonic Science and Technology (PRESTO), Japan Science and Technology Agency (JST) 4-1-8 Honcho Kawaguchi Saitama 332-0012 Japan

## Abstract

The properties of metal nanoclusters depend on both their structures and electronic states. However, in contrast to the significant advances achieved in the synthesis of structurally well-defined metal nanoclusters, systematic control of their electronic states is still challenging. In particular, stimuli-responsive and reversible control of the electronic states of metal nanoclusters is attractive from the viewpoint of their practical applications. Recently, we developed a synthesis method for atomically precise Ag nanoclusters using polyoxometalates (POMs) as inorganic ligands. Herein, we exploited the acid/base nature of POMs to reversibly change the electronic states of an atomically precise {Ag_27_} nanocluster *via* protonation/deprotonation of the surrounding POM ligands. We succeeded in systematically controlling the electronic states of the {Ag_27_} nanocluster by adding an acid or a base (0–6 equivalents), which was accompanied by drastic changes in the ultraviolet-visible absorption spectra of the nanocluster solutions. These results demonstrate the great potential of Ag nanoclusters for unprecedented applications in various fields such as sensing, biolabeling, electronics, and catalysis.

## Abstract 2

The electronic states of Ag nanoclusters were reversibly controlled driven by protonation/deprotonation of polyoxometalate ligands.

## Graphical abstract

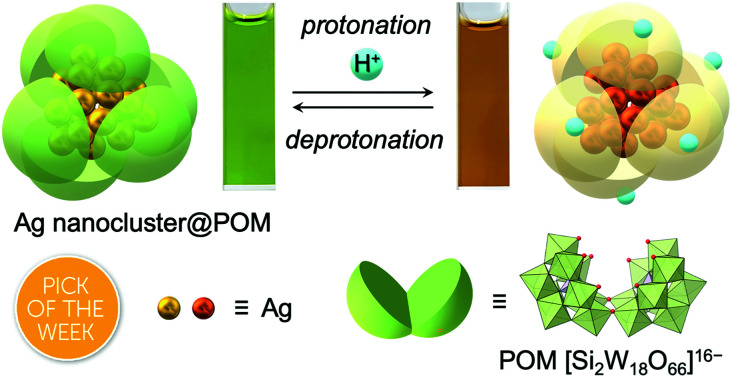

## Introduction

Atomically precise noble metal (typically Au, Ag, Pt, and Pd) nanoclusters have attracted great interest in a wide variety of fields in chemistry, such as photochemistry, electrochemistry, sensing, magnetism, and catalysis, owing to their unique physicochemical properties and reactivities, which strongly depend on their structures, compositions, and electronic states.^1^ Recently, significant advances have been achieved in the development of synthetic methods for atomically precise metal nanoclusters, which rely on the use of organic ligands such as thiolate,^2^ phosphine,^3^ alkynyl,^4^ and carbene ligands.^5,6^ These organic ligands allow not only control of the structures of metal nanoclusters by suppressing undesirable decomposition and aggregation but also modification of their electronic states by electron donation from the ligands to the positively charged nanoclusters. Despite the importance of controlling the transformation of the structures and the electronic states of metal nanoclusters induced by external stimuli for their synthesis and applications, metal nanoclusters that exhibit stimuli-induced reversible transformations are very rare.^7^ This is likely because it is difficult for external stimuli to directly change the properties of the metal nanoclusters in a controlled manner without leading to unfavorable and unpredictable decomposition or aggregation.

The development of a system that enables the control of the electronic states of protecting ligands can be envisaged as a feasible method to achieve stimuli-responsive control of the electronic states of metal nanoclusters. For instance, Negishi *et al.* reported a photoresponsive {Au_25_} nanocluster based on the photoisomerization of a thiol ligand with an azobenzene derivative.^8^ Meanwhile, seeking to exploit protonation/deprotonation as one of the most fundamental and widely available stimuli, Konishi *et al.* reported the control of the electronic states of {Au_8_} nanoclusters *via* the protonation of pyridine-based ligands using excess amounts of acid.^9^ However, in contrast to the significant number of reports on stimuli-responsive Au nanoclusters, their Ag counterparts are more scarce mainly due to their low stabilities.^6*a*,10^ In particular, reversible control of the electronic states of Ag nanoclusters driven by protonation/deprotonation has never been addressed.

We focused on polyoxometalates (POMs) as stabilizing and functionalizing ligands. POMs are anionic metal oxide clusters (typically composed of W^6+^, Mo^6+^, Mo^5+^, and V^5+^) with well-defined structures and unique acid/base, redox, and photochemical properties that can be tuned by modifying their structures, compositions, and countercations.^11^ Therefore, POMs can be fascinating components to design molecular hybrids with organic molecules and metal nanoclusters that exhibit unique properties and applications.^12,13^ In particular, lacunary POMs can act as inorganic multidentate ligands with abundant reactive oxygen atoms.^14^ We recently developed synthetic methods for atomically precise Ag nanoclusters (*e.g.*, {Ag_27_}^17+^ and {Ag_7_}^5+^), which possess valence electrons and superatomic electronic states, using lacunary POMs as inorganic multidentate ligands.^15,16^ These molecular hybrids of Ag nanoclusters and POMs exhibited the following synergetic or cooperative properties, which make them promising for application in a variety of fields: (1) unprecedented high stability even in the solution state; (2) visible-light-responsive charge transfer from Ag nanoclusters to POM frameworks; (3) cooperative dissociation of H_2_ into protons and electrons stored in the Ag nanoclusters and the POM ligands, respectively,^17^ in Ag27 (TBA_16_(Me_2_NH_2_)_8_H_5_Ag_2_[Ag_27_(Si_6_W_54_O_198_)]; TBA = tetra-*n*-butylammonium), in which the {Ag_27_}^17+^ nanocluster is stabilized by three C-shaped [Si_2_W_18_O_66_]^16−^ POM ligands ({Si_2_W_18_}).^15^

**Fig. 1:**
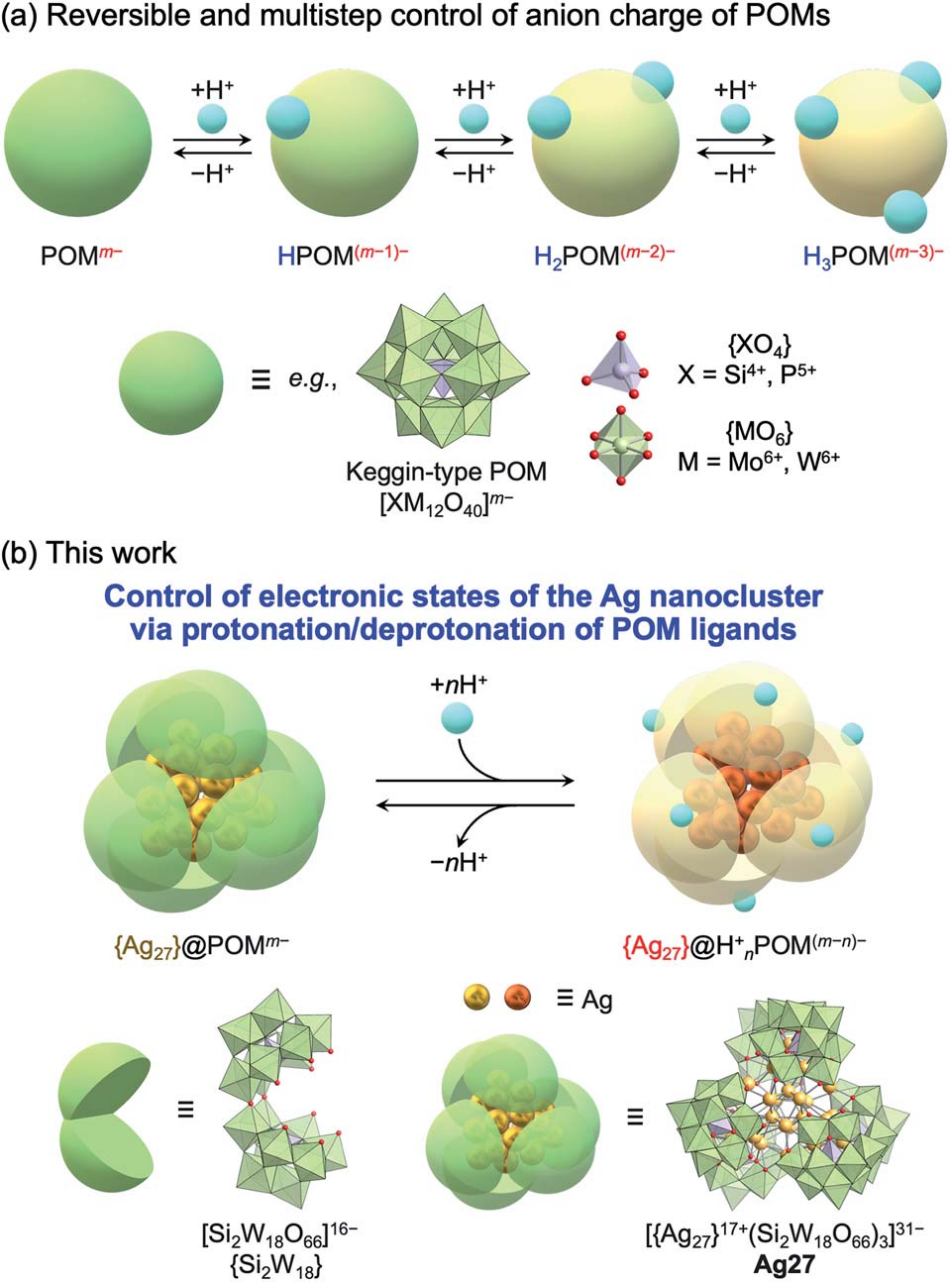
(a) Control of the anionic charge of polyoxometalates (POMs) *via* reversible and multistep protonation/deprotonation. (b) This work: control of the electronic states of an {Ag_27_} nanocluster *via* protonation/deprotonation of [Si_2_W_18_O_66_]^16−^ POM ligands ({Si_2_W_18_}).

Herein, we focused on the unique acid/base properties of POMs, according to which multiple protons can be involved in the protonation/deprotonation of the POM framework by adding acids or bases in organic solvents. Considering that the anion charges and redox properties of POMs can be tuned *via* protonation/deprotonation (Fig. 1a),^18^ we envisaged that the protonation/deprotonation of POM ligands could induce reversible and multistep control of the electronic states and properties of molecular hybrids of POMs and Ag nanoclusters without undesirable structural transformations or decomposition.

In this study, we achieved variable control of the electronic states of the {Ag_27_} nanocluster *via* protonation/deprotonation of the surrounding {Si_2_W_18_} POM ligands in Ag27 (Fig. 1b). The protonation/deprotonation of POM ligands changed the electron donation from the negative [Si_2_W_18_O_66_]^16−^ POM ligands ({Si_2_W_18_}) to the positive {Ag_27_}^17+^ nanocluster, thereby changing the distribution of 10 valence electrons in the nanocluster and leading to drastic changes in the absorption spectra in the visible light region. These results reveal the acid/base properties of POMs as an important tool to control the unique functions and properties of POM-stabilized metal clusters or metal oxide clusters and provide fundamental insights for the development of new applications.

## Results and discussion

**Fig. 2:**
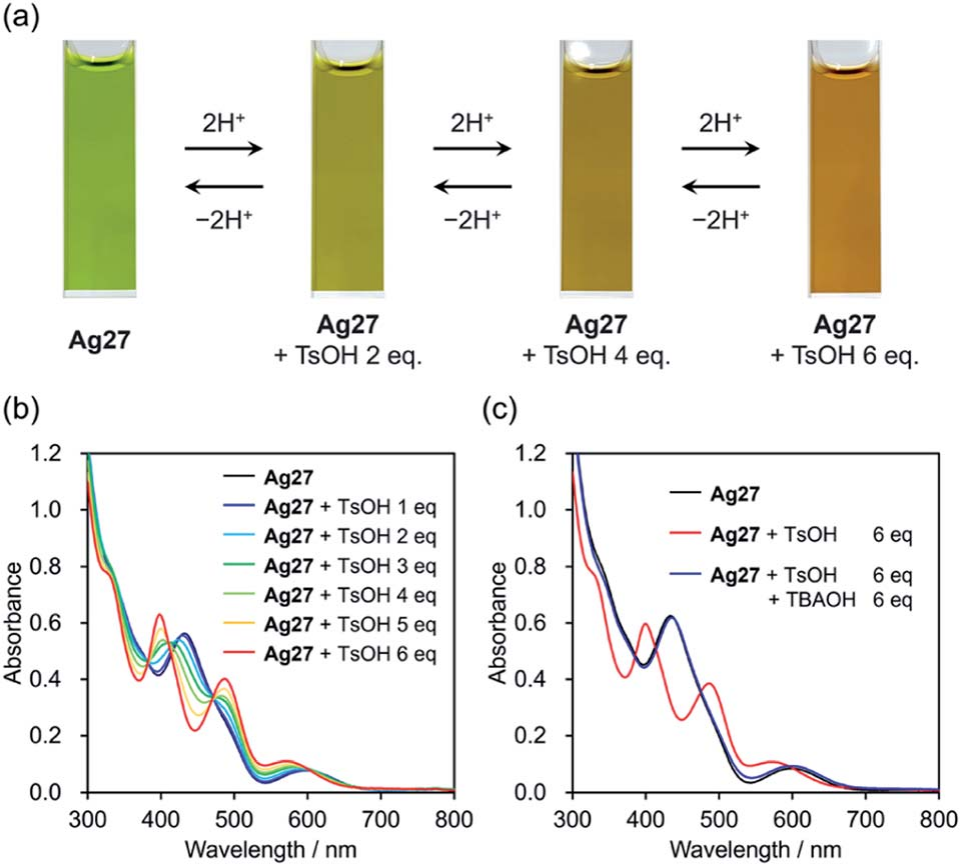
(a) Color change of Ag27 after addition of 0–6 equivalents of *p*-toluenesulfonic acid (TsOH) and tetra-*n*-butylammonium (TBAOH) in acetonitrile. UV-Vis spectra of (b) Ag27 before and after addition of 0–6 equivalents of TsOH and (c) Ag27 before and after addition of 6 equivalents of TsOH and TBAOH in acetonitrile (10 μM, 1 cm cell).

We started our investigation on the protonation/deprotonation of Ag27 by conducting the reaction of Ag27 and *p*-toluenesulfonic acid (TsOH) in acetonitrile at room temperature (∼25 °C). As shown in Fig. 2a, the greenish solution of Ag27 changed to dark red after adding 6 equivalents of TsOH. Before adding the acid, the ultraviolet-visible (UV-Vis) spectrum of Ag27 showed two prominent absorption bands at 440 and 600 nm that can be attributed to the charge transfer from the {Ag_27_} nanocluster-based highest occupied molecular orbital (HOMO) to the POM frameworks (W 5d orbitals) and intra-{Ag_27_} electron excitation.^17^ Upon addition of 2–6 equivalents of TsOH to the acetonitrile solution of Ag27, new absorption bands appeared at 410 and 500 nm with three isosbestic points at 390, 420, and 480 nm, while the absorption band at 440 nm became weaker (Fig. 2b). No changes were observed upon further addition of TsOH beyond 6 equivalents (Fig. S1†). Importantly, by adding 6 equivalents of TBAOH as a base after the addition of 6 equivalents of TsOH, the UV-Vis spectrum of the solution was almost the same as that of the original Ag27, which evidenced that the changes were reversible (Fig. 2c). These results indicated that the molecular structure of Ag27 remained unaltered during the reaction, and no decomposition or aggregation occurred. These changes in the UV-Vis spectra were significantly different from those observed in our previous report on the reaction of Ag27 and H_2_, which induced the reduction of the {Ag_27_} nanocluster and the protonation of the {Si_2_W_18_} POM ligands.^17^

To clarify the changes in the electronic states and structures of Ag and W atoms in Ag27 during the reaction with acids, we subjected acetonitrile solutions of Ag27 and TsOH to an X-ray absorption fine structure study.^19^ The W L_1_-edge X-ray absorption near edge structure (XANES) spectra of Ag27 showed no significant change in the absorption edge energy (*E*_0_ = 12 111 eV) upon addition of 1, 3, and 6 equivalents of TsOH (Fig. S2a†). On the other hand, a slight increase was observed in the intensity of the pre-edge peak around 12 102 eV (Fig. S2b†), which originates from a dipole forbidden 2s → 5d transition for the octahedral {WO_6_} structure. These results indicated that the protonation did not alter the electronic states of the W^6+^ atoms of the {Si_2_W_18_} POM ligands, whereas it decreased the structural symmetry of {WO_6_} octahedral structures. Additionally, the W L_3_-edge XANES spectra showed a slight increase in the intensities of white lines around 10 207 eV upon addition of TsOH. Considering that the W L_3_-edge XANES spectrum is sensitive to structural symmetry as well as electronic states of W 5d orbitals,^19^ these results supported the decrease of the structural symmetry of {WO_6_}, which led to shrinking of d-orbital splitting and increase of peak intensities.

**Fig. 3:**
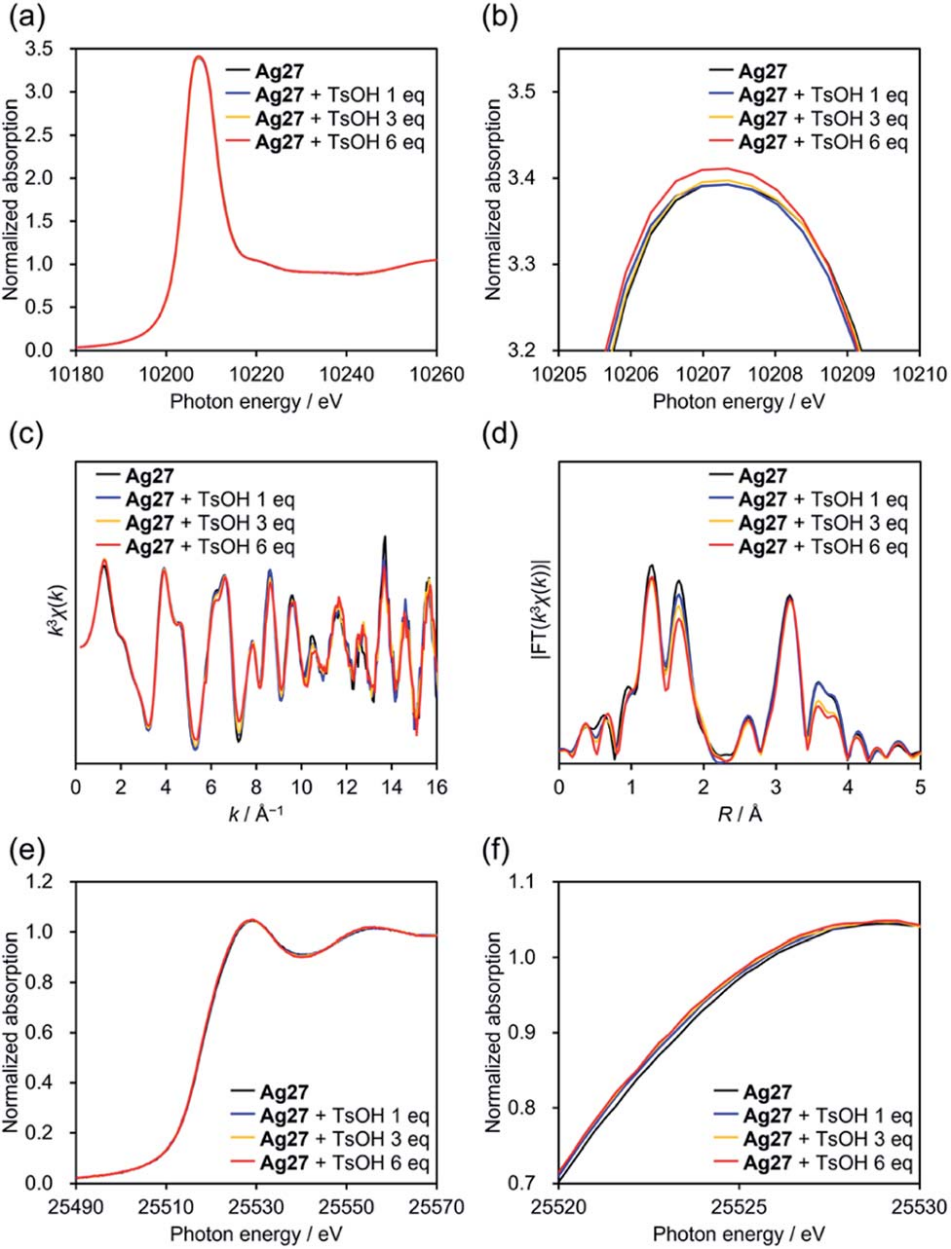
XAFS studies of Ag27 before and after addition of 1, 3, and 6 equivalents of *p*-toluenesulfonic acid (TsOH) in acetonitrile. W L_3_-edge XANES spectra: (a) wide view and (b) enlarged view. (c) *k*-Space EXAFS spectra and (d) Fourier-transformed *R*-space EXAFS spectra (*k* = 3–16 Å^−1^, *k* weight = 3). Ag K-edge XANES spectra: (e) wide view and (f) enlarged view.

We analyzed the W L_3_-edge extended X-ray absorption fine structure (EXAFS) spectra for further investigations on the structure. The W L_3_-edge *k*-space EXAFS spectra showed no significant changes upon protonation, indicating that the {Si_2_W_18_} POM ligands were structurally stable (Fig. 3c). In the *R*-space EXAFS spectra, the peaks at *R* = 1.26 and 3.19 Å assignable to terminal W=O and W⋯W interactions, respectively, exhibited no significant changes after addition of TsOH (0–6 equivalents with respect to Ag27; Fig. 3d), whereas the peak at 1.69 Å attributable to bridging W–O–W decreased. Overall, the reaction of Ag27 and TsOH induced protonation of the O atoms of the {Si_2_W_18_} POM ligands; however, the electronic states of the W atoms hardly changed. Meanwhile, the Ag K-edge XANES spectra underwent a slight shift to the low energy region by adding TsOH, which was indicative of the change in the electronic states of the {Ag_27_} nanocluster upon protonation of Ag27 (Fig. 3e and f). The Ag K-edge *k*-space EXAFS spectra of Ag27 showed no significant changes in the oscillation patterns after the reaction with TsOH, which suggested that the structure of the {Ag_27_} nanocluster intrinsically remained intact (Table S1 and Fig. S3†). In contrast, the fitting analysis of the Ag K-edge *R*-space EXAFS spectra showed that Ag⋯Ag and Ag⋯O distances within the {Ag_27_} nanocluster were slightly modified upon protonation (Table S1 and Fig. S3†).

These results indicated that the electronic states of Ag27 could be tuned by controlling the protonation states of the {Si_2_W_18_} POM ligands. Considering that multistep proton storage in the POM structures allows modifying their negative charge owing to their unique acid/base properties, we speculated that the protonation of Ag27 caused a decrease in the electron donation from the negative O atoms of the {Si_2_W_18_} POM ligands to the {Ag_27_} nanocluster.

**Fig. 4:**
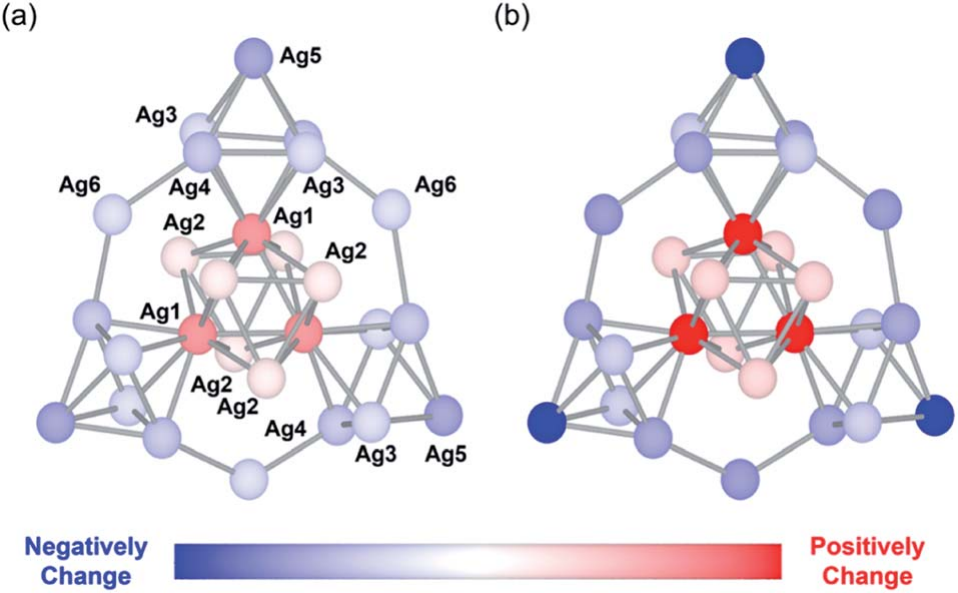
Schematic of the changes in the natural charge of the {Ag_27_} nanocluster of Ag27 upon protonation. Change in the natural charge of each Ag atom after adding (a) three additional protons and (b) six additional protons to the polyoxometalate (POM) frameworks. Silver atoms are colored according to the changes in the natural charges with respect to those of Ag27. The POM frameworks are omitted for clarity (see Fig. S4 in ESI† for the total anion structure of Ag27 including POM frameworks).

Next, we performed density functional theory (DFT) calculations to gain more insight into the unique changes in the electronic states induced by the protonation. Since the electronic states of the {Ag_27_} nanocluster were closely dependent on the distribution of their 10 valence electrons delocalized over the {Ag_27_} nanocluster, we investigated the charge distribution on the Ag atoms according to a natural population analysis. The total natural charges on the {Ag_27_} nanocluster hardly changed upon protonation of the {Si_2_W_18_} POM ligands, being 9.19, 9.18, and 9.18 for Ag27, Ag27 with three additional protons, and Ag27 with six additional protons, respectively. This result indicated that protonation did not alter the total charge of the {Ag_27_} nanocluster. In contrast, the natural charge of each Ag atom clearly changed. The natural charges of the central {Ag_9_} core (Fig. 4; Ag1 and Ag2) increased significantly upon protonation of the {Si_2_W_18_} POM ligands, whereas the natural charges on the three surrounding {Ag_6_} octahedrons (Fig. 4; Ag3, Ag4, and Ag5) and those on the three bridging Ag atoms (Fig. 4; Ag6) decreased. These results showed that the electron density on the central {Ag_9_} core decreased and that on the outer Ag atoms increased. The decrease in the effective anion charge of the {Si_2_W_18_} POM ligands upon protonation likely relieved the repulsion between the 10 valence electrons and the anion charge of the POMs, resulting in an increase in the electron density on the outer Ag atoms close to the POM ligands.

To gain a deeper understanding of the protonation-induced change in the UV-Vis spectra of Ag27, we conducted time-dependent DFT (TD-DFT) calculations (Fig. S5–S7†). According to the TD-DFT calculations of Ag27 having three additional protons and six additional protons on the {Si_2_W_18_} POM ligands, the protonation induced a blue shift of the absorption band at 430 nm to 415 nm likely due to the contribution of the charge transfer from the O atom of the {Si_2_W_18_} POM ligands to the {Ag_27_} core (Fig. S8–S10†). The shoulder peak at 500 nm on the UV-Vis spectra can be assigned to both the intra-{Ag_27_} electron excitation and charge transfer from {Ag_27_} to the W atoms of the POM ligands, mainly through the HOMO−1 orbital. Considering that HOMO−1 resembles a superatomic d-orbital deriving from three surrounding {Ag_6_} octahedrons and a central {Ag_9_} core, the TD-DFT calculation result on protonated Ag27 showing that the absorption band at 500 nm increased upon addition of protons was in agreement with both the experimental UV-Vis absorption spectra and the natural population analysis, according to which the electron density on the three surrounding {Ag_6_} octahedrons increased by adding TsOH.

## Conclusions

In conclusion, we revealed that the electronic states of a POM-stabilized Ag nanocluster could be reversibly changed by controlling the protonation state of the POM ligands. The addition of TsOH decreased the electron donation from the POMs to the Ag nanocluster and consequently changed the electron density distribution on the latter. Noteworthily, this change could be reversibly induced by adding an acid or a base. This study demonstrated the great potential of these molecular hybrids of Ag nanoclusters and POMs in diverse applications such as sensing, bioimaging, and catalysis. Further studies on the unique physicochemical properties of these POM-stabilized Ag nanoclusters, especially external stimuli-responsive properties, and their applications are currently underway in our laboratory.

## Author contributions

K. S. and K. Yo. design the project and experiments. K. Yo. performed the major parts of experiments. S. Y., S. K., K. Yo., K. S. performed XAFS studies. D. Yo. and K. S. performed DFT studies. K. Yo, K. S., and K. Ya cowrote the manuscript.

## Conflicts of interest

There are no conflicts to declare.

## Supplementary Material

SC-013-D2SC01156E-s001
