# Supplementary material for: Variable control of the electronic states of a silver nanocluster via protonation/deprotonation of polyoxometalate ligands
Source: Chem Sci. 2022 Apr 12;13(19):5557–61. doi: 10.1039/d2sc01156e (PMC9116452; doi:10.1039/d2sc01156e)
Supplement: SC-013-D2SC01156E-s001 [file SC-013-D2SC01156E-s001.pdf]

Electronic Supplementary Material (ESI) for Chemical Science.
This journal is © The Royal Society of Chemistry 2022

Electronic Supplementary Information (ESI) for

Variable control of the electronic states of a silver nanocluster by protonation/deprotonation of polyoxometalate ligands

Kentaro Yonesato,[a] Seiji Yamazoe,[b,d] Soichi Kikkawa,[b] Daisuke Yokogawa,[c] Kazuya Yamaguchi*[a] and Kosuke Suzuki*[a,d]

[a] Department of Applied Chemistry, School of Engineering, The University of Tokyo, 7-3-1 Hongo, Bunkyo-ku, Tokyo 113-8656, Japan.
E-mail: ksuzuki@appchem.t.u-tokyo.ac.jp, kyama@appchem.t.u-tokyo.ac.jp
[b] Department of Chemistry, Graduate School of Science, Tokyo Metropolitan University, 1-1 Minami Osawa, Hachioji, Tokyo 192-0397, Japan.
[c] Graduate School of Arts and Science, The University of Tokyo, 3-8-1 Komaba, Meguro-ku, Tokyo 153-8902, Japan.
[d] Precursory Research for Embryonic Science and Technology (PRESTO), Japan Science and Technology Agency (JST), 4-1-8 Honcho, Kawaguchi, Saitama 332-0012, Japan.

Materials: Acetonitrile and *p*-toluenesulfonic acid (TsOH) were obtained from Kanto Chemicals. Tetra-*n*-butylammonium (TBAOH; TBA = Bu_4N^+) was obtained from Sigma-Aldrich. $TBA_{16}(Me_2NH_2)_8H_5Ag_2[Ag_{27}(Si_6W_{54}O_{198})]$ (**Ag27**) was synthesized according to the reported procedure and characterized by ESI-mass spectrometry, UV-Vis spectroscopy, ^{29}Si NMR spectroscopy, and X-ray crystallographic analysis.[S1]

Instruments: Solution-state UV-Vis spectra were measured on a Shimadzu UV-3600 Plus using a quartz cell with the path-length of 1 cm. ESI-mass spectra were measured on a Waters Xevo G2-XS QTof instrument. ^{29}Si NMR spectra were measured on a JEOL JNM ECA-500 spectrometer using 5 mm tubes. Chemical shifts (δ) were reported using $Si(CH_3)_4$ as an internal standard.

X-ray absorption fine structure (XAFS): The Ag K-edge, W L_1- and L_3-edge XAFS measurements were performed at beamline BL01B1 at the SPring-8 facility of the Japan Synchrotron Radiation Research Institute (Proposal number 2021A1272, 2021A1406). The incident X-ray beam was monochromatized by a Si(311) double-crystal monochromator for Ag K-edge measurement and Si(111) double-crystal monochromator for W L_1- and L_3-edge measurement. All XAFS spectra were conducted on the solution of **Ag27** (1 mM acetonitrile solution, 1 cm cell) with addition of TsOH (0.1 mM acetonitrile solution) at room temperature and recorded in transmission mode using ionization chambers. The X-ray absorption near-edge structure (XANES) and extended X-ray absorption fine structure (EXAFS) spectra were analyzed using xTunes program.[S2] Pre-edge background and the EXAFS background were subtracted using McMaster and Cubicspline methods, respectively. The EXAFS spectra were also obtained as k^3-weighted χ spectra after normalization.

Computational details: All calculations were performed with Gaussian 16.[S3] We performed the DFT calculations using CAM-B3LYP functional,[S4] and the solvent effects were included using the polarizable continuum model (PCM, acetone). We employed the moderate-size basis set (6-31G* for O and Si, 6-31G for H, and LANL2DZ ECP for W and Ag) because the size of the target molecule is large. The geometry of **Ag27** used in the calculations was obtained from the crystal structure determined in the reported study. Three and six protons were added to the oxygen atoms of **Ag27** by the following method: To evaluate the protonation effect to the electronic structure, we computed the population change on each atomic site by changing the cavity size used in the PCM calculations. Because **Ag27** has negative charge, the apparent surface charges (ASCs) on the cavity surface are positive and the interaction between **Ag27** and the positive ASCs can mimic the protonation. Based on the population changes along the cavity size change, we determined the protonation positions.

Table S1. Fitting parameters on solution-state Ag K-edge EXAFS for **Ag27** before and after addition of TsOH.[a]

		CN[b]	R^c [Å]	ΔE [eV]	$\sigma^{2\,d}$ [10^{-2}Å^2]	R_f [%]
**Ag27**[e]	Ag…Ag	3.1(3)	2.73(5)	−4.4	1.1(1)	11.9
	Ag…O	0.9(3)	2.25(8)	−9.9	0.4(5)	
**Ag27** + TsOH 1 eq	Ag…Ag	2.4(3)	2.72(4)	−5.4(7)	0.9(1)	6.6
**Ag27** + TsOH 3 eq	Ag…Ag	2.3(3)	2.71(5)	−7.0(7)	0.9(1)	9.4
	Ag…Ag	0.1	3.15	−9.4	0.3	
**Ag27** + TsOH 6 eq	Ag…Ag	2.4(3)	2.72(5)	−5.6(7)	0.9(1)	6.7
	Ag…Ag	0.2(2)	3.20(9)	−10.7(14)	0.3(6)	

[a]k fitting range = 3.0–14.0 Å^{-1}, R fitting range = 2.1–3.2 Å. [b]coordination number; [c]bond length; [d]debye–waller; [e]k fitting range = 1.6–14.0 Å^{-1}, R fitting range = 1.6–3.2 Å

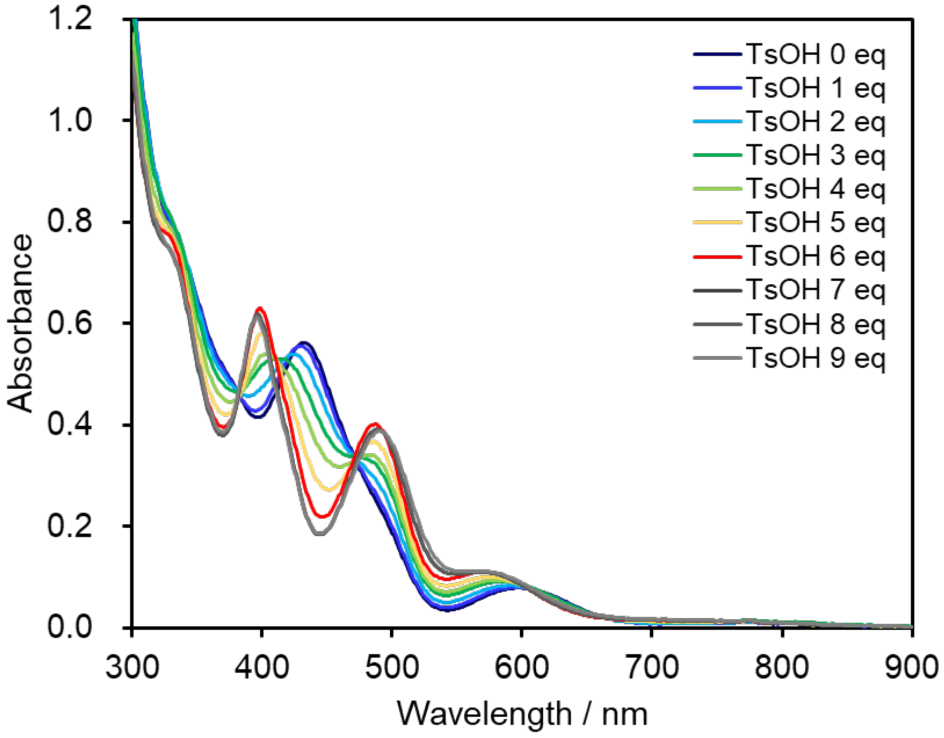

Fig. S1 UV-Vis spectra of **Ag27** before and after addition of TsOH in acetonitrile (10 μM).

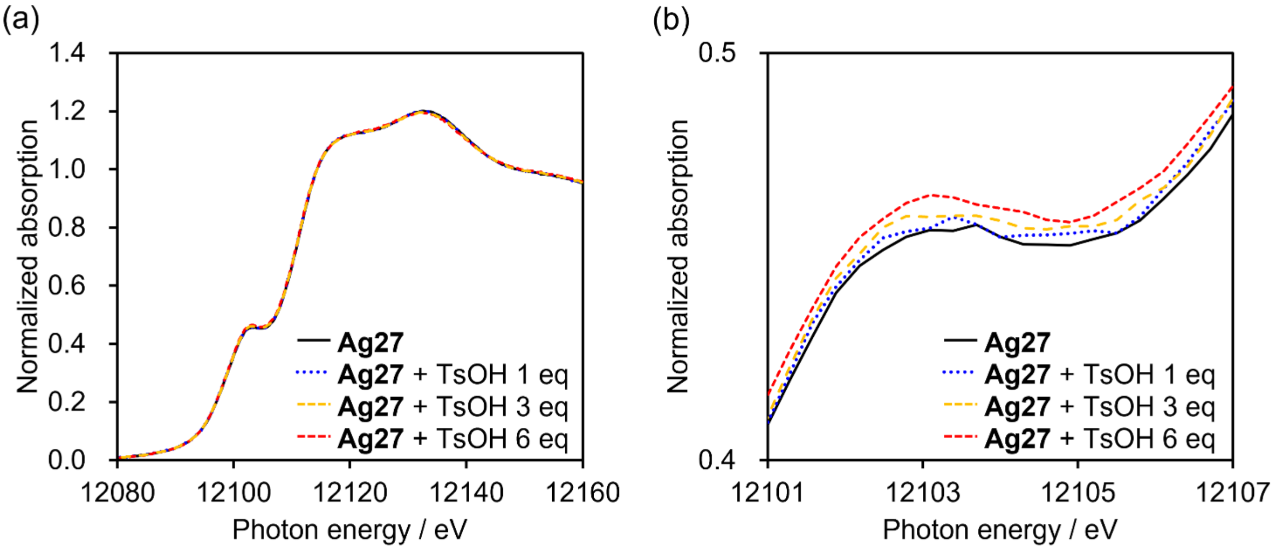

Fig. S2 W L_1-edge XANES spectra of **Ag27** solution in acetonitrile (1 mM) before and after addition of TsOH in (a) wide view and (b) enlarged view around pre-edge peak.

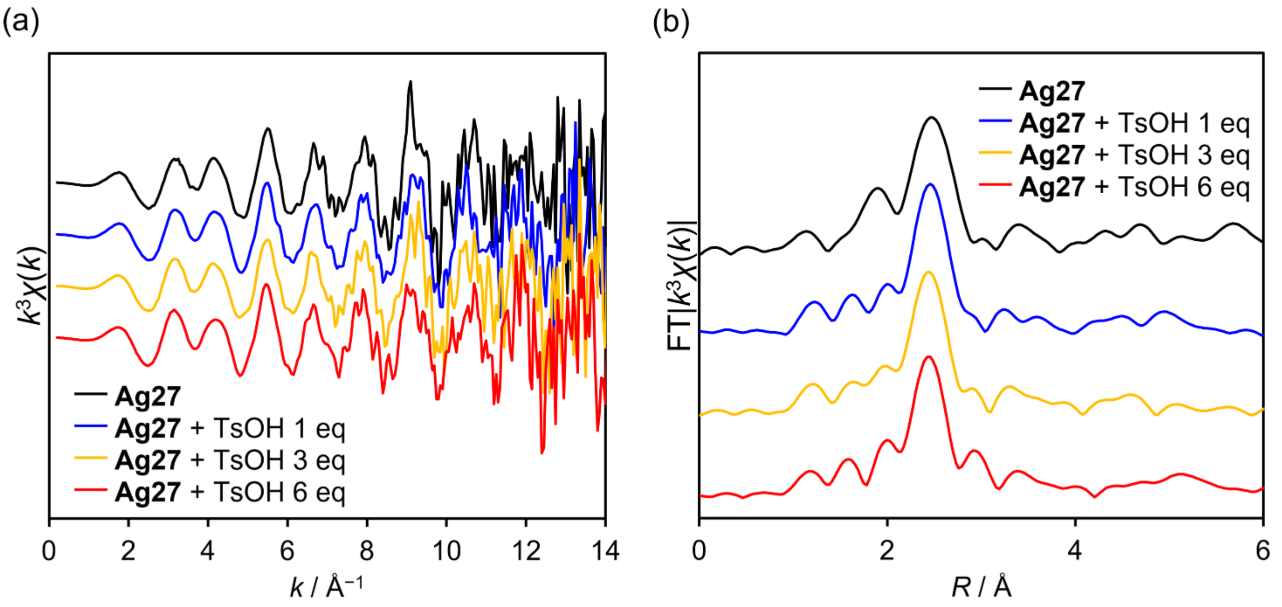

Fig. S3 Ag K-edge (a) *k*-space EXAFS spectra, (b) Fourier-transformed *R*-space EXAFS spectra (k = 3–14 Å^{-1}, k weight =3) of **Ag27** before and after addition of TsOH in acetonitrile.

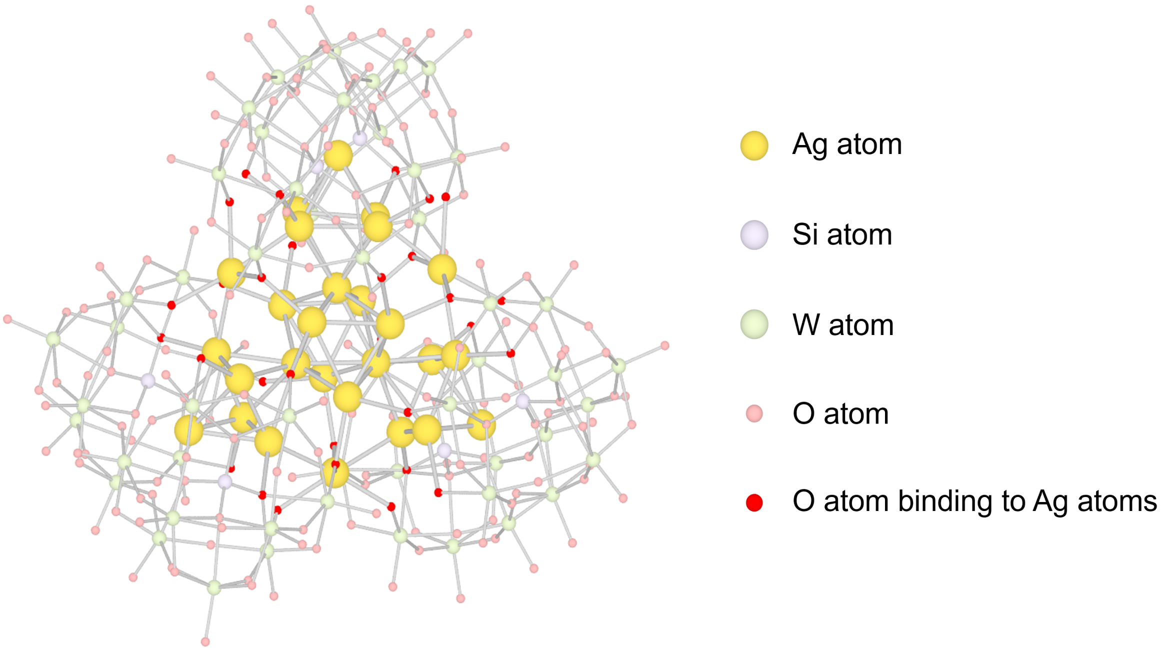

Fig. S4 Anion structure of **Ag27**.

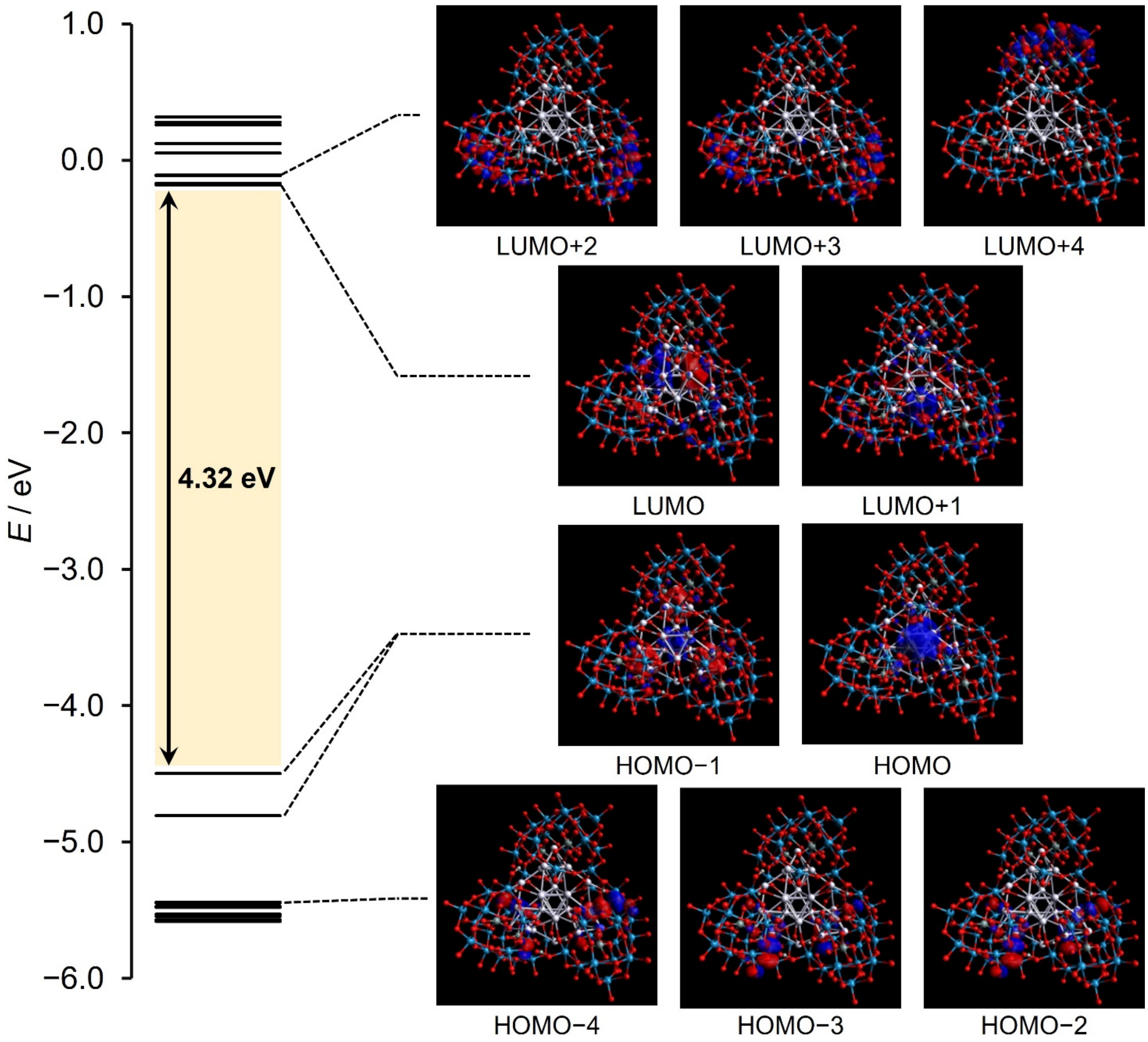

Fig. S5 Energy diagram and molecular orbitals for **Ag27** (6 protons in total) based on the DFT calculation.

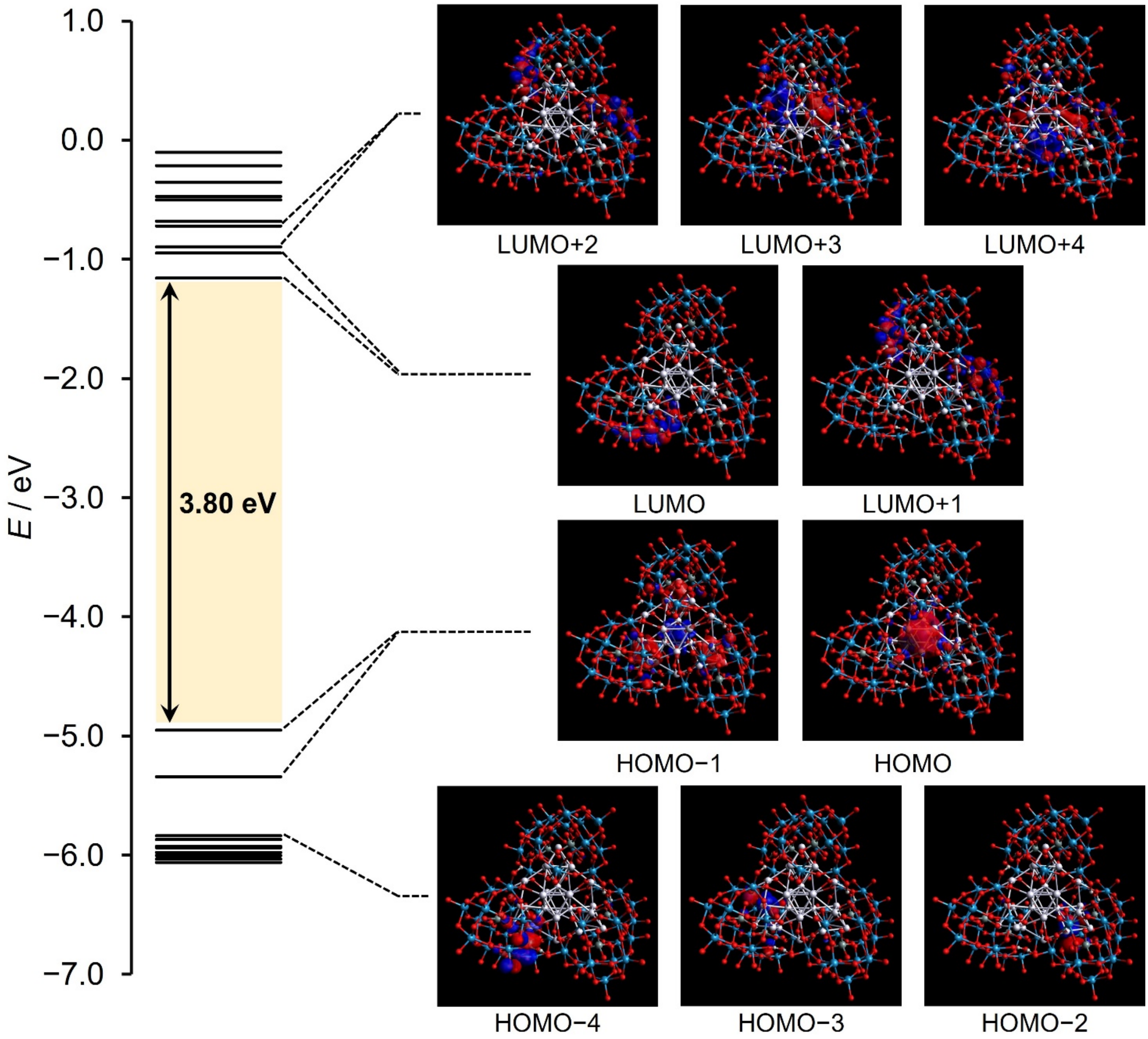

Fig. S6 Energy diagram and molecular orbitals for **Ag27** with additional 3 protons (9 protons in total) based on the DFT calculation.

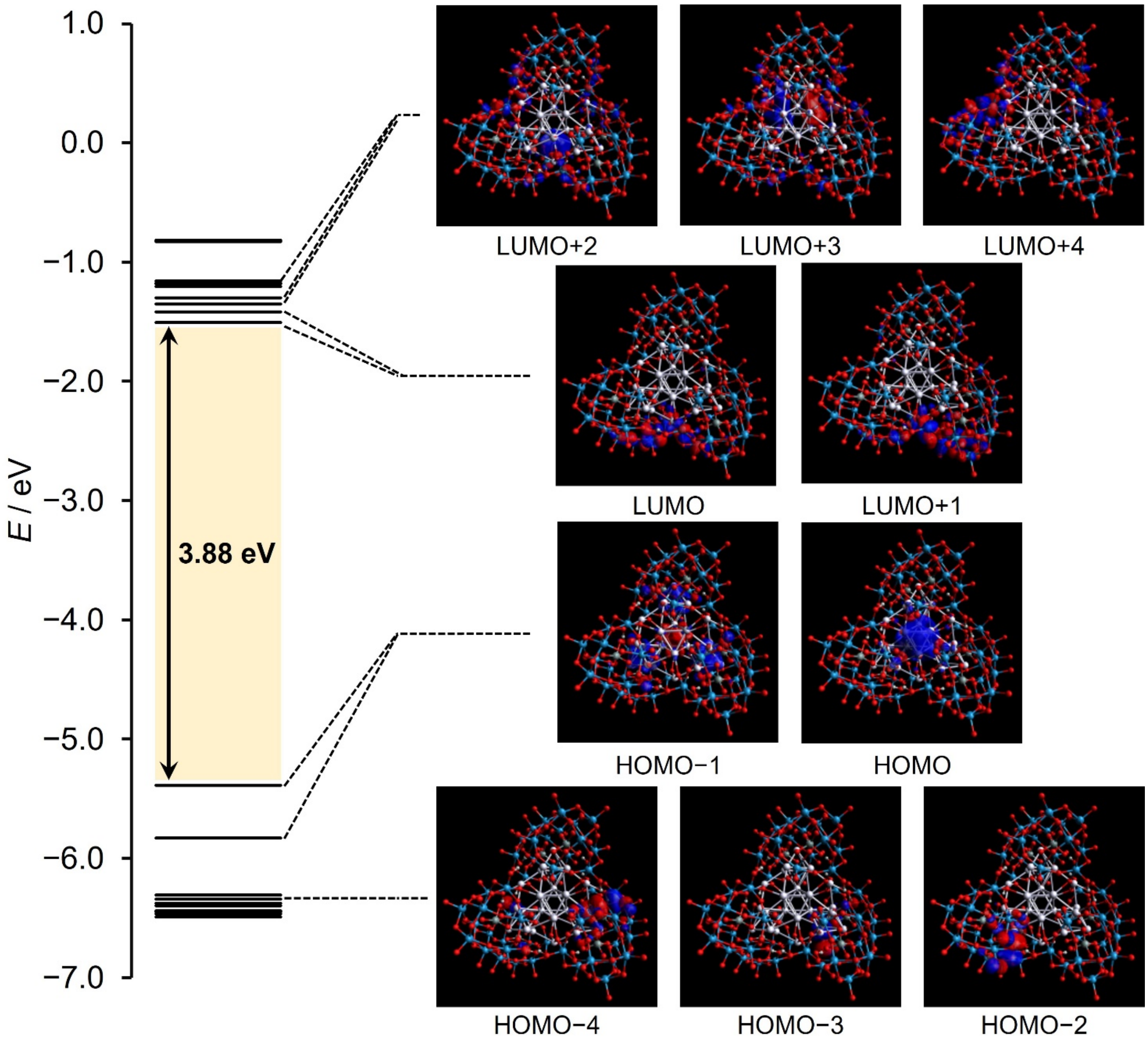

Fig. S7 Energy diagram and molecular orbitals for **Ag27** with additional 6 protons (12 protons in total) based on the DFT calculation.

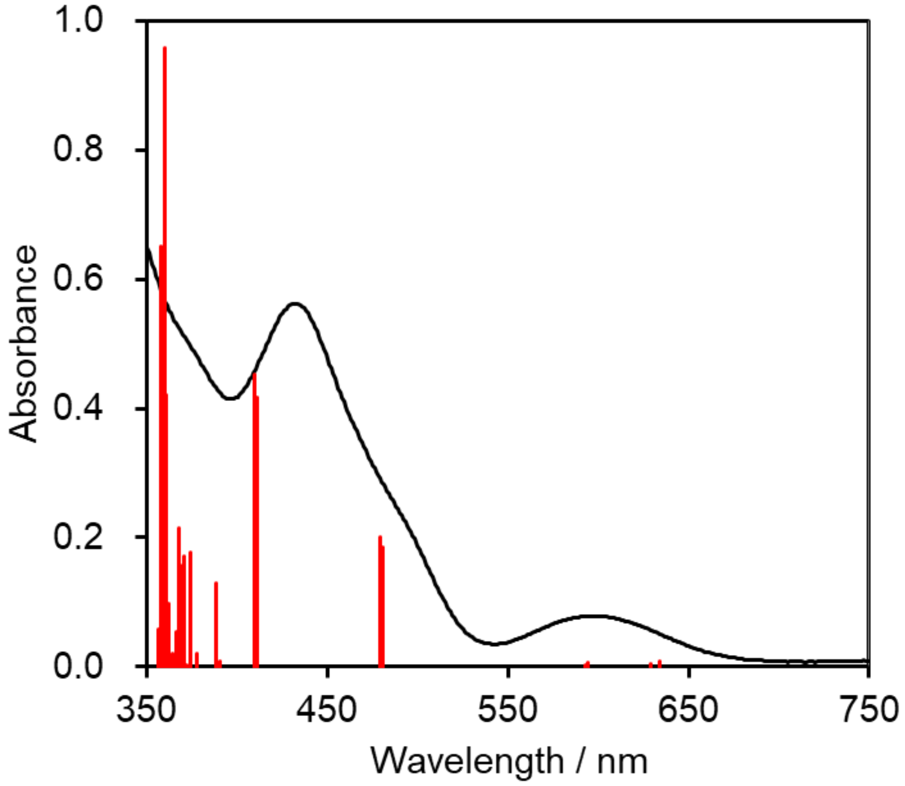

Fig. S8 Experimental UV-Vis spectrum of **Ag27** without addition of protons (black line; acetonitrile, 10 μM) and the calculated transitions for **Ag27** without additional protons based on the TD-DFT calculation (red bars).

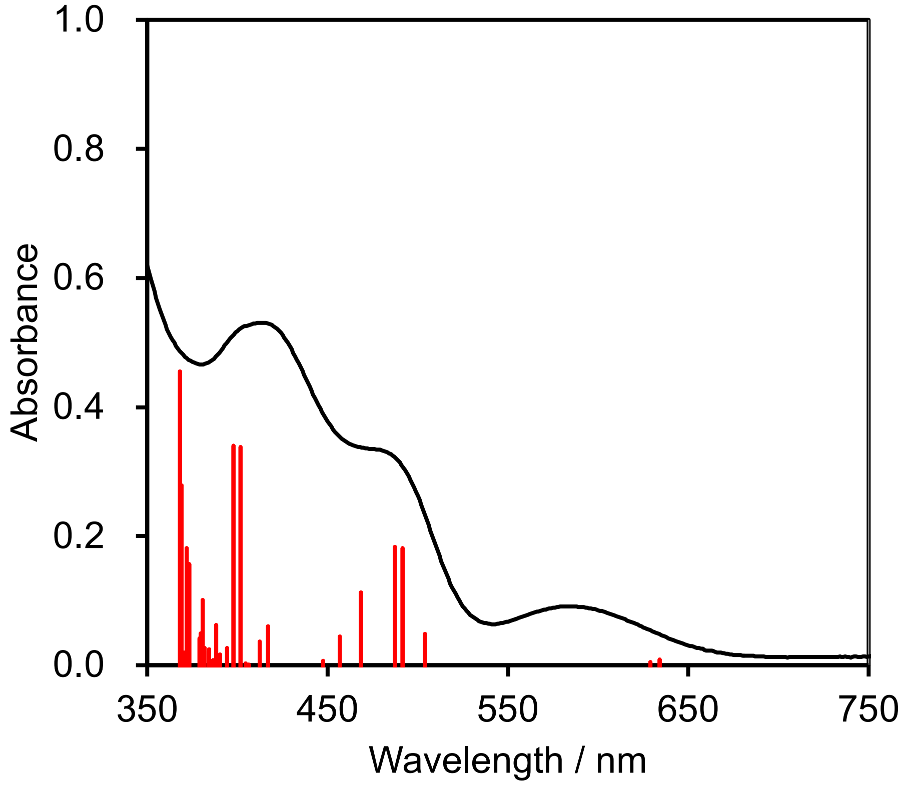

Fig. S9 Experimental UV-Vis spectrum of **Ag27** with addition of 3 equivalents of TsOH (black line; acetonitrile, 10 μM) and the calculated transitions for **Ag27** with additional 3 protons (red bars) based on the TD-DFT study.

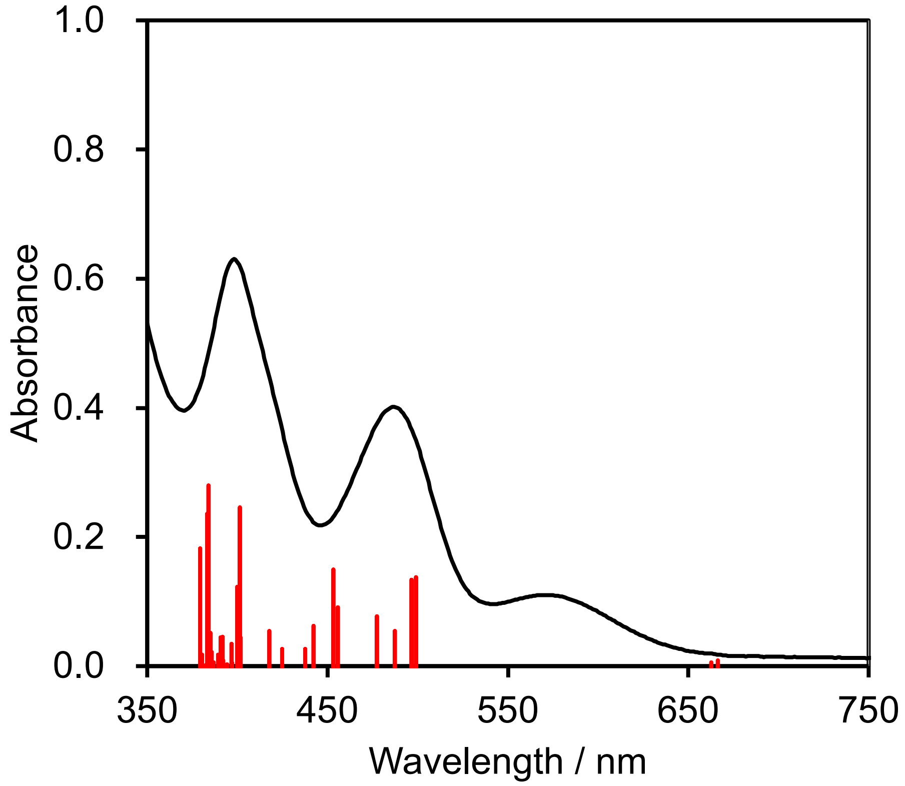

Fig. S10 Experimental UV-Vis spectra of **Ag27** with addition of 6 equivalents of TsOH (black line; acetonitrile, 10 μM) and the calculated transitions for **Ag27** with additional 6 protons based on the TD-DFT calculation (red bars).

References

S1. K. Yonesato, H. Ito, H. Itakura, D. Yokogawa, T. Kikuchi, N. Mizuno, K. Yamaguchi and K. Suzuki, *J. Am. Chem. Soc.*, 2019, **141**, 19550–19554.

S2. H. Asakura, S. Yamazoe, T. Misumi, A. Fujita, T. Tsukuda and T. Tanaka, *Radiat. Phys. Chem.*, 2020, **175**, 108270.

S3. Gaussian 16, Revision B.01, M. J. Frisch, G. W. Trucks, H. B. Schlegel, G. E. Scuseria, M. A. Robb, J. R. Cheeseman, G. Scalmani, V. Barone, G. A. Petersson, H. Nakatsuji, X. Li, M. Caricato, A. V. Marenich, J. Bloino, B. G. Janesko, R. Gomperts, B. Mennucci, H. P. Hratchian, J. V. Ortiz, A. F. Izmaylov, J. L. Sonnenberg, D. Williams-Young, F. Ding, F. Lipparini, F. Egidi, J. Goings, B. Peng, A. Petrone, T. Henderson, D. Ranasinghe, V. G. Zakrzewski, J. Gao, N. Rega, G. Zheng, W. Liang, M. Hada, M. Ehara, K. Toyota, R. Fukuda, J. Hasegawa, M. Ishida, T. Nakajima, Y. Honda, O. Kitao, H. Nakai, T. Vreven, K. Throssell, J. A. Montgomery, Jr., J. E. Peralta, F. Ogliaro, M. J. Bearpark, J. J. Heyd, E. N. Brothers, K. N. Kudin, V. N. Staroverov, T. A. Keith, R. Kobayashi, J. Normand, K. Raghavachari, A. P. Rendell, J. C. Burant, S. S. Iyengar, J. Tomasi, M. Cossi, J. M. Millam, M. Klene, C. Adamo, R. Cammi, J. W. Ochterski, R. L. Martin, K. Morokuma, O. Farkas, J. B. Foresman, and D. J. Fox, Gaussian, Inc., Wallingford CT, 2016.

S4. *(a)* T. Yanai, D. P. Tew and N. C. Handy, *Chem. Phys. Lett.*, 2004, **393**, 51–57; *(b)* T. Tsuneda, J.-W. Song, S. Suzuki and K. Hirao, *J. Chem. Phys.*, 2010, **133**, 174101.
